# Evaluation de la sexualité chez des femmes atteintes d’un cancer du sein après traitement: à propos de 100 cas

**DOI:** 10.11604/pamj.2021.40.38.29406

**Published:** 2021-09-15

**Authors:** Safa Smida, Rim Bouchahda, Ameni Guezguez, Meriem Regaya, Rim Frigui, Ons Kaabia, Hedi Khairi

**Affiliations:** 1 Service de Gynéco-obstétrique, Centre Hôpital Universitaire Farhat Hached de Sousse, Sousse, Tunisie,; 2 Service de Psychiatrie, Centre Hôpital Universitaire Fattouma Bourguiba de Monastir, Tunis, Tunisie,; 3 Ecole Supérieure des Sciences et des Techniques de Santé, Tunis, Tunisie

**Keywords:** Cancer du sein-sexualité image du corps, chimiothérapie, hormonothérapie, radiothérapie, Breast cancer, sexuality, body image, chemotherapy, hormone therapy, radiotherapy

## Abstract

Le cancer du sein est le cancer féminin le plus fréquent. Plusieurs stratégies de traitement sont utilisées : la chimiothérapie, la radiothérapie, la chirurgie et enfin l´hormonothérapie. Chacun de ces traitements est susceptible d´altérer la sexualité des patientes à court ou à long terme. L´objectif de notre étude est d´évaluer la sexualité des femmes atteintes d'un cancer du sein après traitement. Un devis descriptif quantitatif a été réalisé auprès de 100 patientes suivies pour un cancer mammaire non métastasique en activité sexuelle, rencontrées à la consultation de gynécologie de l´hôpital Farhat Hached de Sousse. La collecte des données a été réalisé par une fiche de renseignement et à l´aide de deux échelles validées : RSS (Relation Ship and Sexuality) et BESAA (Body EsteemScale For Adolescents and Adults) afin d´évaluer la sexualité et l´image du corps. La moyenne d´âge était 53,8 ans. Environ la moitié des patientes (48%), avaient une vie sexuelle altérée par la maladie. La fréquence des rapports, le désir sexuel et la capacité d´atteindre l´orgasme a été diminué respectivement chez 65, 45 et 54 patientes. Le score global des 3 dimensions de l´image du corps était de 49,4. Les femmes âgées entre 35 et 39 ans développaient significativement plus de peur du rapport sexuel (p=0,002) et moindre de fréquence sexuelle (p=0,004). Une formation adéquate et accentuée sur la prise en charge des femmes ayant un cancer ainsi que leurs problèmes sexuels et un travail multidisciplinaire permettent d´améliorer l´état psychologique des femmes.

## Abstract (en)

Breast cancer is the most common neoplastic disease in women. Several treatment strategies are used: chemotherapy, radiotherapy, surgery and hormone therapy. Each of these treatments may affect sexual health of patients in the short or long term. The purpose of our study is to assess the quality of sexual life in women after breast cancer treatment. We made a quantitative descriptive estimate of 100 sexual active patients followed up for non-metastatic breast cancer, met during their consultations with a gynaecologist at the Farhat Hached hospital in Sousse. Data collection was carried out using an information sheet and two validated scales: RSS (relation Ship and sexual) and BESAA (Body EsteemScale for adolescents and Adults) to assess the quality of sexual life and body image. The average age of patients was 53.8 years. About half of patients (48%) had impaired sexuality due to the disease. The frequency of intercourse, sexual desire and the ability to reach orgasm were decreased in 65. 45, and 54 patients, respectively. The overall score for the three body image dimensions was 49.4. Women aged between 35 and 39 years were significantly more afraid of sexual intercourse (p=0.002) and less of sexual frequency (p=0.004). Adequate and enhanced training focused on the management of women with cancer and their sexual problems and multidisciplinary approach can improve women’s psychological status.

## Introduction

Le cancer du sein est un problème de santé majeur. Selon l´Organisation mondiale de la santé (OMS); il est le premier cancer chez la femme à la fois dans les pays développés et dans les pays en développement. En Tunisie, le cancer du sein est le cancer féminin le plus fréquent, il représente la première cause de mortalité féminine dans la tranche d´âge de 35 à 55 ans. Entre 800 et 1000 nouveaux cas de cancers du sein sont diagnostiqués chaque année. Cette maladie constitue une préoccupation dans le secteur de santé publique à cause de sa fréquence (20 à 25% des cancers féminins) et du stade clinique au moment du diagnostic avec une fréquence particulière des formes inflammatoires et localement avancé [1]. Plusieurs stratégies de traitement sont utilisées en cas de cancer du sein: la chirurgie de type tumorectomie ou mastectomie, la chimiothérapie, la radiothérapie, et enfin l´hormonothérapie. Les données de la littérature montrent que chacun de ces traitements est susceptible d´altérer la qualité de vie et la sexualité des patientes à court ou à long terme [2]. L´impact de ces traitements sur la sexualité est encore peu étudié auprès des patientes tunisiennes. Dans cette perspective, nous nous sommes proposé de mener une étude dont l´objectif est d´évaluer la sexualité des femmes traitées pour cancer du sein en rémission et décrire la nature des dysfonctionnements.

## Méthodes

**Type et lieu d´étude**: il s´agit d´une étude transversale descriptive, menée durant une année (2019) à la consultation de gynécologie de l´hôpital Farhat Hached de Sousse.

**Population cible**: notre population cible était composée de patientes suivies pour un cancer mammaire non métastasique en activité sexuelle. Notre recherche a été effectuée sur un échantillon non probabiliste par convenance regroupant 100 patientes. Le recrutement des patientes a été effectué aux consultations dans le cadre du suivi des contrôles post thérapeutiques. Les patientes recrutées avaient un carcinome mammaire prouvé histologiquement non métastasique, dont l´âge était inferieur ou égale à 60 ans et leur traitement adjuvant à base de chimiothérapie et /ou radiothérapie était terminé depuis au moins trois mois et actuellement, elles sont en rémission complète de leur cancer avec une vie sexuelle active et un partenaire sexuel présent.

**Recueil des données**: avant la réalisation de notre travail, on a expliqué aux patientes les motifs de l´étude. La collecte des données a été réalisée après leurs consentements à l´aide d´un questionnaire préétabli en langue arabe. Pour la réalisation de l´étude une fiche de recueil de données pour chaque patiente a été remplie qui a comporté: Les caractéristiques sociodémographiques des patientes et de leurs partenaires (L´âge, lieu de résidence, le niveau d´instruction et l´activité professionnelle pour la patiente et son mari). Les données cliniques (statut ménopausique, les caractéristiques de la tumeur : stade TNM) et thérapeutiques (le traitement chirurgical (type, reconstruction mammaire), la chimiothérapie (type, durée, tolérance), la radiothérapie (type, durée, tolérance), l´hormonothérapie (type, durée, tolérance), le traitement ciblé (type, durée, tolérance)), ont été recueillies à partir des dossiers des patientes.

**Instruments de mesure**: la sexualité et l´image du corps ont été évaluées en utilisant pour l´évaluation de la sexualité l´échelle RSS (Relation Ship and Sexuality) [3] et pour l´image du corps l´échelle BESAA (Body-EsteemScale For Adolescents and Adults) [4].

**Echelle Relationship and sexual scale (RSS)**: l´échelle RSS comprend 19 items répartis comme suit: a) cinq items définissant la dimension « dysfonctionnement sexuel » et qui sont les items 2, 3, 4, 11, 12; b) trois items explorant la dimension « fréquence sexuelle » et qui sont les items 8, 13,16; c) deux items formant la dimension « peur sexuelle » qui sont les items 9 et 10, neuf items en lien avec la sexualité et qui sont 1, 5, 6, 7, 14, 15, 17,18 et 19. Les scores pour chaque item varient de (0 à 3) ou de (0 à 4).Un score moyen global est obtenu par le calcul de la moyenne de toutes les réponses. Un score moyen a été également calculé pour chaque dimension et pour chaque item séparément. Ces scores moyens permettent, lors de l´étude analytique, la comparaison des sous-groupes. Pour chaque item, on a considéré que la sexualité est négativement affectée pour les valeurs suivantes:

**Le dysfonctionnement sexuel**: score global pathologique ≥13; Item2: score de 2 à 3; Item3: score =2; Item4: score =2; Item11: score=3; Item12: score=3.

**Fréquence sexuelle**: score global pathologique ≤4; Item 8: score ≤2; Item13: score≤2; Item16: score=0.

**Peur sexuelle**: score global pathologique de 4 à 8; Item9: score de 2 à 4; Item 10: score de 2 à 4.

**Les autres items**: pathologiques si: Item 1: score=0; Item5: score de 0 à 2; Item 6: score de 3 à 4; Item7: score de 0 à 2; Item14: score=0; Item15: score de 3 à 4.

### Echelle Body- Esteem Scale for Adolescent and Adults BESAA

L´échelle BESAA permet de mesurer l´image du corps elle comporte 23 items regroupés en 3 dimensions: a) la perception de l´apparence: BE-apparence: estime générale par rapport à l´apparence (items: 1, 6, 7, 9, 11, 13, 15, 17, 21, 23); b) l´attribution: BE-Attribution: la perception d´un individu de l´évaluation des autres par rapport à son corps et son appence (items: 2, 20, 12, 5, 14); c) le poids: la satisfaction vis-à-vis du poids corporel: Be-weigt (items: 3, 4, 8, 10, 16, 18, 19,22). **Chaque item est coté sur une échelle de 5 point allant de 0 à 4**: a) l´astérisque connote les items dont les notes doivent être inversées (0=4, 1=3,2=2,3=1); b) le score varie de 0 à 92 et à seulement une valeur dimensionnelle .Plus le score est élevé, meilleure est l´estime de l´image corporelle.

**Saisie et analyse des données**: la saisie et l´analyse des données ont été réalisées à l´aide du logiciel SPSS (version 21) et présentées sous forme des tableaux. Nous avons utilisé le test t student pour la comparaison de deux moyennes, le test ANOVA pour la comparaison de plusieurs moyennes et la corrélation bi-varié pour la comparaison des variables quantitatives. La valeur p< 0.05 était considérée comme statistiquement significative.

**Considérations éthiques**: nous avons expliqué l´objectif de notre étude, son but purement scientifique et le contenu des questions aux patientes avant la collecte des données. De même elles ont été informées du caractère anonyme et de la confidentialité des informations retenues.

## Résultats

### Caractéristiques de la population étudiée

La population globale étudiée est constituée de 100 patientes. Ces femmes ont une moyenne d´âge de 53,8 ans. Concernant la fertilité des participantes, 10% des femmes avaient un problème d´infertilité qu´à l'inverse, 90 % avaient des enfants (en moyenne 2 enfants à leur charge). Dans notre étude 55% des femmes étaient d´origine urbaine. Quarante pourcent de ces femmes avaient un niveau universitaire. Plus que la moitié (65%) des interrogées vivent seules dans leurs maisons. Trente Cinq pourcent des participantes sont des femmes au foyer. Au moment de l´étude 22% des femmes étaient en congés de maladie et 28% avaient repris leur travail. Pour les partenaires, on note que 32% occupent un poste de fonctionnaire. Plus que la moitié des participantes (57%) étaient en ménopause. Au moment du diagnostic de cancer, le stade tumoral le plus fréquent était le stade T2 observé chez 52% des patientes (Tx=2%, T1=15%, T3= 20%, T4b=7%, T4d=4%) et le creux axillaire était libre (pas d´atteinte ganglionnaire = N0) dans 75% des cas (Nx=2%, N1=15%, N2=8%).

Le traitement chirurgical était radical de type mastectomie dans 58% des cas et conservatrice de type tumorectomie dans 32% des cas. Dix pourcents des femmes avaient bénéficié d´une reconstruction mammaire après la chirurgie. La majorité des patientes (85%) ont bénéficié d´une chimiothérapie. La tolérance à la chimiothérapie était mauvaise chez 30% des patientes et chez 47%, elle était moyenne. Soixante et onze pourcents des patientes ont bénéficié d´une radiothérapie. Soixante-huit pourcents avaient rapporté une bonne tolérance à la radiothérapie et seulement 5% avaient rapporté une mauvaise tolérance. Environ la moitié des participantes (48%) ont reçu une hormonothérapie adjuvante. L´hormonothérapie était à base de tamoxifène dans 33% des cas, des anti-aromatases dans 27% des cas et l´association de deux traitements dans 40% des cas. Pendant la période d´étude, l´hormonothérapie était non encore achevée chez 60% des patientes. Les 2/3 (60%) des femmes avaient bien toléré leur traitement hormonal et 20% l´avaient mal toléré. Environ 1/3 des femmes (33%) avaient reçu une thérapie ciblée de type Herceptin et 40% entre elles étaient en cours de traitement pendant la période d´étude. Ces femmes avaient une bonne tolérance vis-à-vis l´Herceptin.

### Étude de la sexualité

En ce qui concerne le dysfonctionnement sexuel, presque la moitié des patientes (48%) avait une vie sexuelle altérée par la maladie. En effet, la fréquence des rapports, le désir sexuel (RSS3 et RSS 4) et la capacité d´atteindre l´orgasme (RSS 12) a diminué respectivement chez 65, 45 et 54 patientes. L´insatisfaction du mode de la relation affective dans le couple était trouvée chez 75 patientes (RSS 8 et RSS 13) et 15 femmes avaient affirmé qu´elles n´ont pas eu de rapports sexuels pendant les deux dernières semaines. Pour la dimension peur sexuelle, 20 patientes avaient rapporté une peur des rapports sexuels. Concernant les connaissances des patientes vis-à-vis le sujet, la quasi-totalité des participantes (90%) a affirmé qu´elle n´avait pas reçu d´informations à propos l´impact de cancer de sein sur la relation sexuelle (RSS1). Trente pourcent des patientes et de leurs partenaires avaient été émotionnellement séparés au cours de la maladie (RSS 6), tandis que 25% des femmes étaient émotionnellement rapprochées de leurs partenaires. Plus que la moitié des patientes (55%) se sentaient moins attirantes sexuellement dont 22% souffraient d´une sécheresse vaginale. Le score global des 3 dimensions de l´image du corps était de 49,4 avec un minimum de 32 et maximum de 70.

### Étude analytique

La relation entre les troubles sexuels et les paramètres sociodémographiques est étudié dans le Tableau 1. Les femmes âgées entre 35 et 39 ans développaient significativement plus de peur du rapport sexuel (p=0,002) et moindre de fréquence sexuelle (p=0,004). Cependant, nous n´avons trouvé aucune association significative entre l´activité professionnelle et les troubles sexuels. L´analyse de la relation entre les troubles sexuels et les données clinique est résumée dans le Tableau 2. Nous n´avons trouvé aucune association significative entre les troubles sexuels et la ménopause, la taille tumorale ainsi que le statut ganglionnaire. L´étude de la relation entre les troubles sexuels et les données thérapeutiques sont résumés dans le Tableau 3. Les troubles sexuels n´étaient pas significativement corrélés ni à la chirurgie, ni à la chimiothérapie ni à la thérapie ciblée, tandis que l´hormonothérapie était significativement associée à l´altération du fonctionnement sexuel (p=0,008) et à une moindre fréquence sexuelle (p=0,01). La relation entre les troubles sexuels et l´image du corps est résumé dans le Tableau 4. La perception de l´apparence et la satisfaction vis-à-vis le poids étaient significativement associé à la peur sexuelle (p=0,008 vs p=0,01).

**Tableau 1:** relation entre les troubles sexuels (mesuré par l´échelle RSS) et les paramètres sociodémographiques chez un groupe de 100 femmes atteintes d´un cancer du sein après traitement et suivi à l´hôpital Farhat Hached Sousse

Variables	Les troubles sexuels					
Paramètres Sociodémographiques	Dysfonctionnement sexuel	P	Fréquence sexuel	P	Peur sexuelle	P
**Age**		0,09		0,004		0,002
35-39 ans	12,1±1		5,1±1,3		2±0,5	
40-49 ans	9,02±1,8		6,2±1,8		0,5±0,1	
50-60 ans	11,2±1,2		3,99±1,1		0,41±0,12	
**Profession**		0,88		0,72		0,2
Sans profession	10±1,9		5±1,8		0,51±0,5	
Ouvrière	10,5±1		5,4±1,7		0,48±0,4	
Fonctionnaire	9±2,1		6,9±2		0,3±0,5	
Cadre supérieur	11,1±2,9		4,2±3,9		1,3±1,2	

**Tableau 2:** relation entre les troubles sexuels (mesuré par l´échelle RSS) et les données cliniques chez un groupe de 100 femmes atteintes d´un cancer du sein après traitement et suivi à l´hôpital Farhat Hached Sousse

Données clinique	Les troubles sexuels					
	Dysfonctionnement sexuel	p	Fréquence sexuel	p	Peur sexuelle	p
**Ménopausée**		0,62		0,11		0,79
**Oui**	9,9±1,1		4,1±0,5		0,5±0,3	
**Non**	10,1±0,8		5,2±0,8		0,61±0,2	
**Taille tumorale**		0,19		0,3		0,1
Tx	11		5		0	
T1	10±1,1		6±0,5		0,2±0,3	
T2	8±0,4		5,9±1,9		0,5±0,12	
T3	11±1,8		3,1±2		1±0,5	
T4b	12,3±1		4,5±2,8		1,8±0,9	
T4d	9,8±1,2		6,1±0,5		0	
**Ganglions**		0,49		0,52		0,54
Nx	11		5		0	
N0	10±1		10,5±1,1		0,92±0,3	
N1	5,7±0,2		6,1±0,5		0,5±0,1	
N2	0,8±0,1		0,3±0,1		1,2±1	

**Tableau 3:** relation entre les troubles sexuels (mesuré par l´échelle RSS) et les données thérapeutiques chez un groupe de 100 femmes atteintes d´un cancer du sein après traitement et suivi à l´hôpital Farhat Hached Sousse

Données thérapeutiques	Les troubles sexuels					
	Dysfonctionnement sexuel	p	Fréquence sexuel	p	Peur sexuelle	P
**Chirurgie**		0,13		0,4		0,6
Mastectomie	10,1±0,4		6,1±0,3		0,5±0,1	
Tumerctomie	11,1±0,5		5±0,7		0,97±0,2	
**Chimiothérapie**		0,19		0,29		0,91
Mauvaise tolérance	12,3±0,9		2,6±0,5		1±0,5	
Tolérance moyenne	9,7±1		6,7±0,1		0,6±0,32	
Bonne tolérance	8,5±1		4,9±1		0,8±0,15	
**Hormonothérapie**		0,008		0,01		0,2
Oui	11,4±0,5		5,1±0,2		0,5±0,11	
Non	9±0,1		5,9±0,3		1,1±0,4	
**Hormonothérapie en cours**		0,1		0,31		0,005
Oui	9±1		6,1±1,01		0,8±0,4	
Non	8±0,74		6,88±0,58		0,2±0,1	
**Radiothérapie**		0,12		0,3		0,98
Oui	9,77±0,7		5,86±0,6		0,77±0,2	
Non	8,1±0,8		6,9±1		0,15±0,1	
**Tolérance de radiothérapie**		0,06		0,15		0,5
Mauvaise tolérance	6±1		10±2,1		0	
Tolérance moyenne	8,99±0,7		6±0,5		0,66±0,31	
Bonne tolérance	10,4±0,5		5,1±0,5		0,7±0,1	
**Herceptin**		0,1		0,5		0,11
Oui	10±0,32		6,1±0,5		0,21±0,1	
Non	10,2±0,89		4,99±0,46		1	
**Herceptin en cours**		0,94		0,67		0,12
Oui	10,1±0,5		5,7±0,52		0,4±0,13	
Non	10,03±0,49		5,13±1		0,88±0,21	

**Tableau 4:** relation entre les troubles sexuels (mesuré par l´échelle RSS) et l´image du corps (mesurée par l´échelle BESAA) chez un groupe de 100 femmes atteintes d´un cancer du sein après traitement et suivi à l´hôpital Farhat Hached Sousse

Variables	Les troubles sexuels					
Image du corps	Dysfonctionnement sexuel	p	Fréquence sexuel	p	Peur sexuelle	p
Perception de l´apparence	10,28±0,66	0,9	6,18±0,81	0,09	0,52±0,1	0,008
Satisfaction vis-vis du poids	10,17±0,49	0,4	4,7±0,4	0,3	0,77±0,21	0,01
Evaluation positive attribuée aux autres	9±1,2	0,22	6,4±0,53	0,1	0,92±0,18	0,33

## Discussion

La prise en charge de cancer du sein chez notre population comprenait divers traitements appropriés à cette maladie: la chirurgie était radicale (mastectomie) chez 58% des patientes et conservatrice (tumorectomie) chez 42% des patientes. Dix pourcents de l´ensemble de la population avaient bénéficié d´une reconstruction mammaire. Les études récentes suggèrent l´avantage de la chirurgie conservatrice en matière de répercussions sur la sexualité et que la mastectomie, (avec ou sans reconstruction), conduit à une réduction significative du confort avec le partenaire en postopératoire par rapport à la tumorectomie [5,6]. La chimiothérapie était prescrite pour 85% des patientes. En outre, la chimiothérapie chez ces patientes avait une tolérance moyenne dans 47% des cas. Plusieurs études ont confirmé l´influence de la chimiothérapie sur la sexualité, puisqu´elle induit une aménorrhée prolongée, une sécheresse vaginale, une diminution du désir, de l´excitation sexuelle et une difficulté d´atteindre l´orgasme. Les femmes jeunes sont plus susceptibles de développer des dysfonctionnements sexuels en raison des conséquences physiques et psychologiques [7,8].

Nos résultats indiquent qu´environ la moitié des participantes (48%) ont reçu une hormonothérapie adjuvante et 40% d´entre elles n´ont pas encore achevées leur traitement. En outre, les 2/3 des femmes avaient bien toléré leur traitement hormonal. D´après Fourati *et al*., l´hormonothérapie a été associée à des difficultés d´excitation, d´orgasme et à la dyspareunie [9]. A travers la littérature plusieurs études ont exploré les répercussions du traitement du cancer sur la sexualité, des troubles sexuels ont été rapportés. Les auteurs ont pu cerner certains facteurs prédictifs d´une sexualité plus ou moins harmonieuse [10]. Presque la moitié de nos patientes (48%) avait une vie sexuelle altérée par la maladie. En effet, la fréquence des rapports, le désir sexuel (RSS3 et RSS 4) et la capacité d´atteindre l´orgasme (RSS12) ont diminués respectivement chez 65, 45 et 54 patientes. On comparant nos résultats à celles objectivés dans l´étude d´Ellouze, on trouve que dans cette dernière la prévalence de la dysfonction sexuelle peut atteindre 75 % des cas. Une diminution du désir sexuel était notée chez 56 % de femmes, une diminution de l´excitation sexuelle chez 60 % et une difficulté à atteindre l´orgasme chez 59 % [11]. Ces valeurs sont proches de celles trouvées dans notre étude à l´exception d´une prévalence de dysfonction sexuelle plus élevée dans l´étude d´Ellouze par rapport à notre étude.

L´insatisfaction du mode de la relation affective dans le couple était trouvée chez 75 de nos patientes (RSS 8 et RSS 13) et 15 autres avaient affirmé qu´elles n´ont pas eu de rapports sexuels pendant les deux dernières semaines. En ce sens une étude tunisienne a objectivé un changement de la fonction sexuelle des partenaires après le diagnostic de cancer du sein dans 38,3% des cas. Cette même étude a démontré l´effet des difficultés sexuelles du partenaire dans l´insatisfaction sexuelle des femmes tunisiennes atteintes de cancer du sein [12]. Pour la dimension peur sexuelle, 20 patientes avaient rapporté une peur des rapports sexuels. Selon une étude française réalisée auprès de 378 femmes traitées pour un cancer de sein non métastasique et évaluées par l´échelle RSS, 38 % des femmes ont rapportés une peur sexuelle et 29% ont rapporté l´absence des rapports sexuels pendant les 2 semaines précédant l´enquête [13]. Dans notre étude, la quasi-totalité des participantes (90%) ont affirmées qu´elles n´avaient pas reçu d´informations à propos l´impact du cancer du sein sur la relation sexuelle (RSS1). Ceci en similitude avec l´étude Jeannin *et al*. en 2012 qui indique que les personnels de santé ont évoqué des freins à donner des informations sur le cancer et à l´accès à un accompagnement psychologique (et en particulier l´absence de besoin ou de demande). Il était donc nécessaire, non seulement, que le patient ressente le besoin pour entamer le besoin en informations et l´accompagnement psychologique, mais également qu´il en fasse la demande [14].

Concernant la relation du couple, 30% de nos patientes et de leurs partenaires avaient été émotionnellement séparés au cours de la maladie (RSS 6). Un changement au niveau de la dynamique du couple a été noté chez 68,08% des cas dans l´étude de Mnif *et al*. en 2016 [12]. L´altération de la relation de couple a été identifiée par Ellouze *et al*. en 2018 comme facteur lié à la dysfonction sexuelle chez les femmes atteintes de cancer du sein [11]. Plus que la moitié de nos patientes (55%) se sentait moins attirantes sexuellement dont 22% souffraient d´une sécheresse vaginale. Zaeid *et al*. ont rapporté que la dyspareunie et la sécheresse vaginale était présente chez 45% patientes dans leur étude [15]. Le score global des 3 dimensions de l´image du corps était de 49,4. Il parait que nos enquêtées ont préservé une image du corps après le traitement du cancer du sein. Dans ce cadre plusieurs études ont démontré que l´altération de l´image du corps augmente significativement la prévalence de la dysfonction sexuelle chez les femmes atteintes de cancer du sein [15,16]. Dans le même contexte, la littérature montre que la mastectomie est une étape violente physiquement et psychologiquement. En effet, cette dernière est ressentie très négativement car elle représente un bouleversement de l'image du corps et une atteinte à l'intégrité corporelle féminine. En effet, les médecins doivent accompagner les femmes et expliquer le déroulement des soins [17]. Dans notre étude, on a trouvé que les femmes âgées entre 35 et 39 ans développaient significativement plus de peur du rapport sexuel (p=0,002) et moindre de fréquence sexuelle (p=0,004). Cependant, nous n´avons trouvé aucune association significative entre l´activité professionnelle et les troubles sexuels. Cour. F et Bonierbale. M en 2013 affirment que la construction du désir sexuel féminin est éminemment multifactorielle. Savoir situer un trouble du désir sexuel dans l´histoire de la patiente, dans son contexte médical et relationnel, est donc essentiel. Sans désir, la femme jeune peut ne pas assimiler l´absence de sexualité à une souffrance. Ce sont souvent les conséquences de cette situation qui l´amèneront à consulter. Les motifs les plus fréquents de consultation sont soit la crainte de ne pas être comme les autres femmes ou la peur de perdre son partenaire [18].

Les troubles sexuels n´étaient pas significativement corrélés ni à la chirurgie, ni à la chimiothérapie ni à la thérapie ciblée, tandis que l´hormonothérapie était significativement associé à l´altération du fonctionnement sexuel (p=0,008) et à une moindre fréquence sexuelle (p=0,01). La littérature indique que l´hormonothérapie a pour effet de diminuer la croissance des cellules cancéreuse en empêchant l´organisme de produire certaines hormones. Cette hormonothérapie peut avoir des répercussions sur la vie sexuelle des patientes à cause des effets secondaires qu´elle engendre comme la fatigue, la perte de libido ou encore les nausées et les vomissements. En utilisant l´hormonothérapie comme traitement de leur cancer, la majorité des femmes sont en mesure d´éprouver du plaisir et d´atteindre l´orgasme tout en suivant cette thérapie. Ce traitement provoque parfois la ménopause qui n´est pas nécessairement permanent et par conséquence la sécheresse vaginale qui est un effet secondaire susceptible d´affecter le plaisir sexuel [19]. La perception de l´apparence et la satisfaction vis-à-vis le poids étaient significativement associé à la peur sexuelle (p=0,008 vs p=0,01). L´impact du cancer et de ses traitements sur la vie sexuelle varie en fonction de chacun. Le désir sexuel régresse bien souvent avec des conditions de stress plus importantes. L'anxiété quant à l'évolution de la maladie et les conséquences des traitements anticancéreux, comme les changements d'apparence physique, la fatigue, des perturbations de l'équilibre hormonal; peuvent entraîner une baisse importante de la libido [20].

**Limites de notre étude**: a) le refus de certaines patientes à participer à l´étude; b) le flux des malades dans les consultations de carcinologie a été une difficulté lors de la collecte des résultats.

## Conclusion

Le cancer du sein est un problème de santé majeur qui prend de plus en plus d'ampleur dans notre société. Ces traitements ne sont pas sans conséquences compte tenu qu'ils touchent un des symboles de la maternité et de la féminité. Par conséquent, ils touchent la sexualité en provoquant chez certaines femmes des perturbations psychologiques et physiques. Parmi ces répercussions on peut citer la perturbation de l'image corporelle, de l'image de soi et le dysfonctionnement sexuel. De ce fait, une formation adéquate et accentuée sur la prise en charge des femmes ayant un cancer ainsi que leurs problèmes sexuels et un travail multidisciplinaire (Médecin, infirmier, sage-femme, psychologue, conjoint) permettent d´améliorer l´accompagnement de ces femmes et par conséquent l'état psychologique. Ce qui permet une meilleure adaptation face à leur sexualité.
